# Dehydrated Alfalfa and Fresh Grass Supply in Young Rabbits: Effect on Performance and Caecal Microbiota Biodiversity

**DOI:** 10.3390/ani9060341

**Published:** 2019-06-11

**Authors:** Simona Mattioli, Alessandro Dal Bosco, Sylvie Combes, Livia Moscati, Silvia Crotti, Alice Cartoni Mancinelli, Elisa Cotozzolo, Cesare Castellini

**Affiliations:** 1 Department of Agricultural, Environmental and Food Science, University of Perugia, Borgo XX Giugno 74, 06121 Perugia, Italy; alessandro.dalbosco@unipg.it (A.D.B.); acartonimancinelli@gmail.com (A.C.M.); elisa.cotozzolo@libero.it (E.C.); cesare.castellini@unipg.it (C.C.); 2 GenPhySE, Université de Toulouse, INRA, ENVT, 31300 Toulouse, France; sylvie.combes@inra.fr; 3 Institute Zooprofilattico Umbria and Marche, Via Gaetano Salvemini 1, 06126 Perugia, Italy; l.moscati@izsum.it (L.M.); s.crotti@izsum.it (S.C.)

**Keywords:** rabbit, peri-weaning feeding, caecal microbiota, dehydrated alfalfa, fresh grass

## Abstract

**Simple Summary:**

The weaning of young rabbits is a critical period that is often accompanied by digestive troubles. Innovations in feeding strategy are urgently needed to preserve rabbit health and to reduce the use of antibiotics. We show here that providing dehydrated alfalfa during weaning is a promising solution to manage health status by favoring the establishment of a proper digestive microbiota.

**Abstract:**

The improvement of rabbit gut microbiota by modifying nutritional components of the feed or favoring its early intake of feed has been previously investigated. The early administration of dehydrated alfalfa (A) or fresh grass (G) for rabbits, during the peri-weaning period (30 and 45 days of age), and their effect on performance and caecal microbiota compared to a standard diet (C) were evaluated. Until 15 days of age, nine litters/group were housed in the maternal cage and milked once per day. From 15 to 30 days, the young rabbits could consume both milk and solid feed (pelleted for C or supplemental feed for A and G). At 30 days of age, the rabbits were weaned and, until 45 days, were kept in single cages following the same dietary protocol. No significant changes were found in the milk intake or the individual weight of young rabbits at 30 and 45 days. The caecal Firmicutes/Bacteroidetes (bacterial phyla ratio) increased with age (from 2.43 to 6.05 on average, at 30 and 45 days). The Ruminococcaceae/Lachinospiraceae (bacterial family ratio) was highest in the A group at both ages, followed by G then C. The early administration of dehydrated alfalfa is a promising solution to improve health status by favoring an appropriate digestive microbiota.

## 1. Introduction

The high incidence of digestive disease during the post-weaning period of young rabbits is a relevant problem worldwide; the etiology of digestive diseases is poorly understood and is often related to an unbalanced microbiota composition [1]. Due to this, anti-microbials are frequently used as therapy or disease prevention to limit the occurrence of these digestive troubles [2]. However, the use of such anti-microbials in animal production is critically viewed because of their impact on the development of resistant bacteria [3]. There are many documented cases of the transmission of antibiotic resistance from animals to humans and the environment [4].

Studies in poultry [5,6], pigs [7], and rabbits [8,9] highlighted the importance of microbiota for digestive health and the immune system. The control of microbiota might also have a direct impact on the cost of production by limiting digestive problems around weaning due to the selective barrier effect, through its role as an immune stimulator and by reducing post-weaning mortality. One promising approach is to manipulate the microbiota of the gut to optimize rabbit health by modifying nutritional components of the feed [10,11,12] or by favoring the early intake of feed. Combes et al. [13] suggested that, during the initial colonization stages of the gut, the rabbit microbiota is unstable and undergoes microbial changes until 21–35 days of age; moreover, during the pre-pubertal period, the microbiota of the mother can influence and support the stabilization of the intestine [14]. Furthermore, Gidenne et al. [15] suggested that the feed limitation (in terms of duration period, intensity, or method) in the post-weaning period is a successful strategy to improve the digestive physiology of rabbit, because the slower passage of feed results in a more efficient digestion. The authors reported that the reduction of the voluntary intake up to 40% or the access time to the feeders improved the feed conversion and the digestive health status in adult rabbits; these results are partially due to changes in digestive physiology (higher villi area and height) [16] and in immune system response (increased lymphocyte number) [17].

In the light of what was reported, the aim of the present study was to analyze two dietary strategies (the early administration of dehydrated alfalfa or fresh grass), and to evaluate their effect on the performance and caecal microbiota structure of rabbits during the peri-weaning period.

## 2. Materials and Methods

### 2.1. Animals and Experimental Design

The trial was carried out at the experimental farm of the Instituto Zooprofilattico Sperimentale dell’Umbria e delle Marche, according to EU 2010/63/EU and Italian 26/2014 directives. Specific experimental authorization was given by the Ethical Committee, number 397/2016-PR on 26 April 2016. The environmental temperature and relative humidity were controlled (ranges: 18–27 °C and 60–75%, respectively). The building was artificially ventilated (0.3 m^3^/s).

Twenty New Zealand White rabbit does were inseminated and 12 homogeneous multiparous pregnant does were selected for the trials. A semi-intensive reproductive rhythm (interval between parturition was 42 days) was applied. At birth, litters were homogenized to nine young rabbits/doe. Fifteen days after the kindling, does and litters were divided into three groups (four doe–litters/group):

- Control group (C); does and young rabbits were fed standard pelleted feed (alfalfa meal 38 g/100 g, barley 19 g/100 g, maize gluten feed 15 g/100 g, extruded soybean 14 g/100 g, wheat bran 7 g/100 g, and vitamin and mineral mix 7 g/100 g).
- A group; does and young rabbits were fed the same standard pelleted feed as group C but had additional access to dehydrated alfalfa (furnished by Carli, Pietracuta di San Leo; RN, Italy; in blocks of size 3 cm length × 2 cm width × 1 cm thickness).
- G group; does and young rabbits were fed the same standard pelleted feed as group C but had additional access to fresh grass (cut every day at an average height of 20 cm). The main species of the natural pasture were the following: *Arrhenatherum elatius*, *Brachypodium* spp., *Dactylis glomerata*, *Lolium* spp., *Lotus cornicolatus*, *Onobrycis viciaefolia*, *Pisum sativum*, *and Capsella bursa-pastoris*.

Does were housed in World Rabbit Science Association (WRSC) cages modified by adding a hay feeder in the nest and reducing the nest door dimensions. The dimensions of the cage were 38 cm width (W) × 1030 cm length (L) × 60 cm high (H) in the highest part of the cage and 35 cm in the lowest part. The nest was 38 cm W × 25 cm L × 35 cm H. In the nest was added a hay feeder of 25.4 cm H × 25.4 cm L, to provide both pelleted and supplemental feed. The mothers did not have access to the nest feeder during the experimental period, because the nest door was reduced in dimension using a wire mesh.

**Figure 1:**
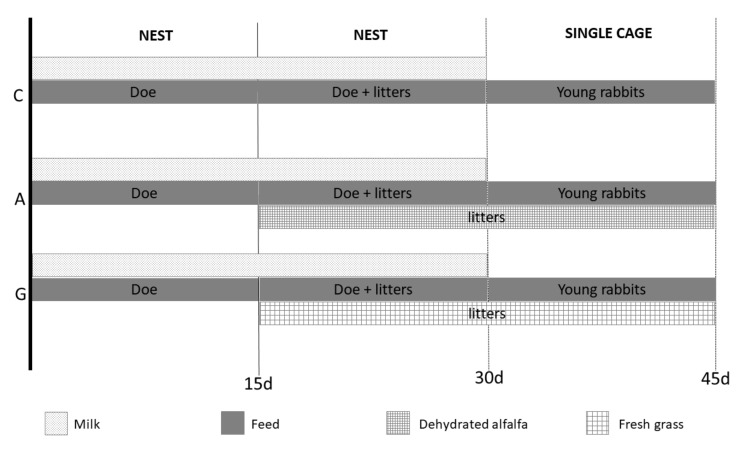
Feeding plan of experimental groups. The dotted bar represents the milking period; the full gray bar represents the pelleted feed administration; the small square bar represents the dehydrated alfalfa period of administration; the big square bar represents the fresh grass period administration. C = rabbit fed pelleted feed only; A = rabbit fed pelleted feed + dehydrated alfalfa; G = rabbit fed pelleted feed + fresh grass.

The dietary experimental plan is shown in Figure 1.

From the day of birth until 15 days of age, litters were housed in the nest of the maternal cage and milked once per day (7:00–7:30 a.m.), after which the nest was closed. From 15 to 30 days of age, the young rabbits had free access to the mother’s cage and could consume both milk and solid feed. To monitor the feed intake, two feeders were installed in the cage: one inside the nest for exclusive use by the litter, and the other one in the main part of cage for mother and litters; as previously reported in groups A and G, alfalfa and fresh grass were also furnished, respectively. At 30 days of age, young rabbits were weaned and were kept in single cages (dimensions: 38 cm W × 60 cm L × 35 cm H) following the same dietary protocol until 45 days (C: only solid feed; A: solid feed plus alfalfa; G: solid feed plus fresh grass).

Throughout the entire trial, water, pelleted feed, dehydrated alfalfa, and fresh grass were provided *ad libitum*.

### 2.2. Productive Performance

Peri-weaning mortality and feed intake (solid pelleted diet, dehydrated alfalfa, and fresh grass) were recorded daily. No data were recorded on water intake. Litters were weighed daily until weaning age (30 days), whereas, from 30 to 45 days, rabbits were individually weighed. Milk production was evaluated by weighing litters before and after access to milk (0 to 15 days). The feed efficiency from 30 to 45 days was also calculated.

### 2.3. Slaughtering and Sampling

At 30 and 45 days of age, 10 young rabbits/group were randomly selected, weighed, and sacrificed in accordance with the 2010/63/EU directive transposed into the 26/2014 Legislative Decree by an overdose of pentobarbital sodium at a dose of 200 mg/kg administered intravenously. The gastro-intestinal tract and cecum were immediately removed.

### 2.4. Analytical Evaluations

#### Proximate Composition of Feed and Supplemental Diets

Dry matter was determined by oven-drying at 105 °C overnight [18]. Crude protein was measured by a Kjeldahl nitrogen analysis [18]. Lipids were extracted by diethyl ether using a Soxhlet apparatus (SER 148, VELP Scientifica, Monza-Brianza, Italy). Ash content was determined by combusting for 3 h at 550 °C. Crude fiber was determined as described by Reference [18]. Neutral detergent fiber (NDF), acid detergent fiber (ADF), and acid detergent lignin (ADL) content was determined according to Reference [19]. The total intake (pelleted and supplemental feed) of all fiber components (NDF, ADF, ADL, hemicellulose, and cellulose) was also calculated.

### 2.5. Caecal Microbiota Evaluation

#### 2.5.1. DNA Extraction

Total genomic DNA from approximately 0.2 g of caecal sample was extracted and purified using the QIAamps DNA Stool Mini kit (Qiagen Ltd., West Sussex, UK) according to the manufacturer’s instructions after thermal (in liquid nitrogen) and mechanical (bead beating using 400 mg of 0.1-mm glass beads) lysis.

#### 2.5.2. 16S Ribosomal RNA (rRNA) Gene Sequencing

The V3–V4 regions of 16S rRNA genes in the DNA extract from the caecal samples were amplified from purified genomic DNA using the primers forward F343 (5′–CTTTCCCTACACGACGCTCTTCCGATCTTACGGRAGGCAGCAG–3′) and reverse R784 (5′–GGAGTTCAGACGTGTGCTCTTCCGATCTTACCAGGGTATCTAATCCT–3′). The PCR was carried out with an annealing temperature of 65 °C for 30 amplification cycles. At the Genomic and Transcriptomic Platform (INRA, Toulouse, France), single multiplexing was performed using 6-bp index sequences, which were added to R784 during a second PCR with 12 cycles. The resulting PCR products were purified and loaded onto the Illumina MiSeq cartridge (Illumina, San Diego, CA, USA) according to the manufacturer’s instructions. Each pair-end sequence was assigned to its sample with the help of the previously integrated index.

#### 2.5.3. Sequence Analysis

A total of 4116 16S ribosomal DNA amplicon sequences were sorted based on their respective barcodes, representing the 60 caecal samples. Using FROGS [20], in keeping with the standard operating procedures (SOP), sequences were filtered by removing sequences that did not match both proximal PCR primer sequences (no mismatch allowed), had an erroneous sequencing length (<400 or >500 nucleotides), or had at least one ambiguous base. Chimeric DNA sequences were detected using VSEARCH and removed. Reads were clustered into operational taxonomic units (OTUs) using SWARM [21]. OTU taxonomic assignment was performed using the basic local alignment search tool (BLAST) algorithm against the SILVA SSU Ref NR 128 database [22].

### 2.6. Statistical Analyses

The statistical analysis of the productive performance of the rabbit does and young rabbits was performed using a mixed linear model (STATA-MIXED) [23] with the fixed effect of feed treatment and accounting for the repeated measurements with litter as a random effect. The significance of differences in Least Square (LS) means was assessed using a *t*-test (*p* < 0.05).

The statistical analyses of all other traits (microbiota analysis) were carried out using R software, version 3.4.2 [24], in RStudio software, version 1.1.383 [25]. Shannon and InvSimpson diversity indexes were calculated, and the structure of the bacterial community was investigated after calculation of a Bray–Curtis distance matrix that was plotted using a principal coordinate analysis, after matrix rarefaction normalization. To check for group differences, an ADONIS pairwise test with the Bray–Curtis distance was carried out. At the taxonomic level, significant differences between feeding groups and the two ages were reported, but their interaction (feed × age) was not significantly different.

## 3. Results

### 3.1. Proximate Composition of Pelleted and Supplemental Feed

The alfalfa had a higher fiber content than fresh grass, of which the main form was NDF, followed by ADF and ADL. Accordingly, the cellulose and hemicellulose content was also higher (Table 1).

### 3.2. Productive Performance of Does, Litters, and Young Rabbits

No significant differences were found between the weight of individual litters at both 30 and 45 days of age (Table 2).

In both the experimental groups (A and G), the milk intake of the litter was not significantly different than control (Table 2). The pre-weaning mortality was the same (12.5%) in all the groups, and no death occurred after weaning.

**Figure 2:**
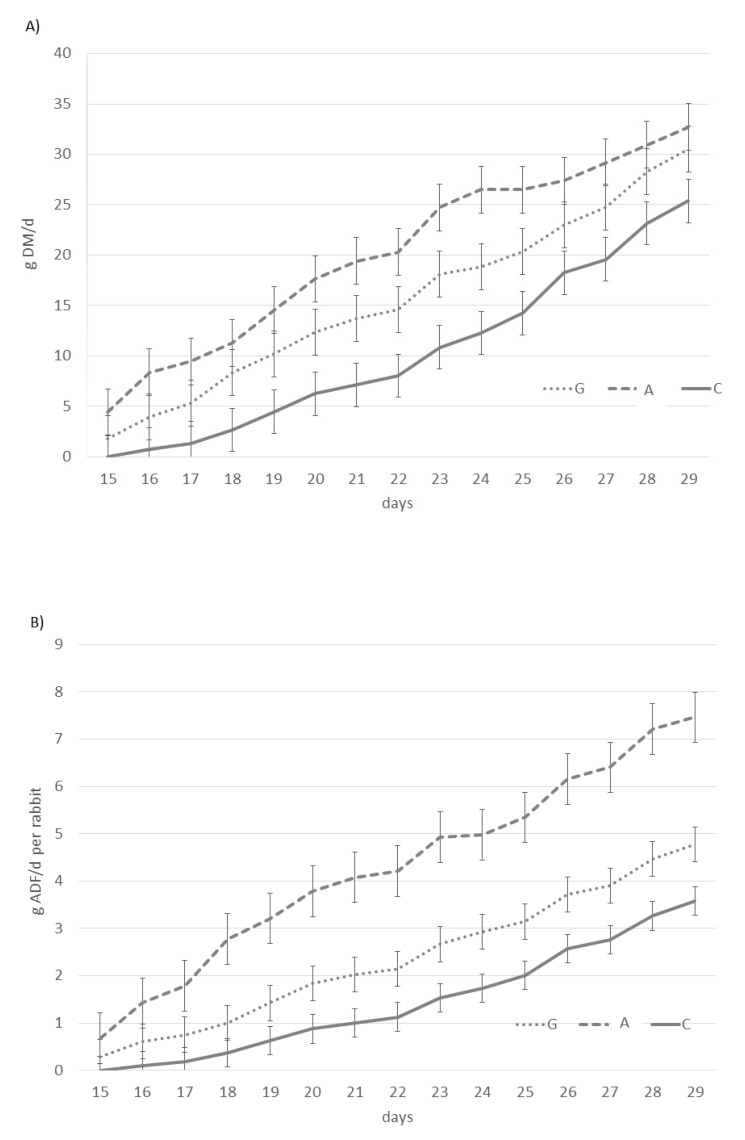
Total feed intake on a dry matter (DM) basis (**A**) and acid detergent fiber (ADF) intake (**B**) of young rabbits from 15 to 30 days of age. The dotted line represents rabbit fed pelleted feed + fresh grass (G group); the dashed line represents rabbit fed pelleted feed + dehydrated alfalfa (A group); the solid line represents rabbit fed pelleted feed (C group).

The feed intake of group G (feed + fresh grass) during milking was higher than that of group A (feed + alfalfa) and the control, when expressed on a fresh matter basis (data not shown); however, on a dry matter (DM) basis, the A intake was higher compared to that of G and C (Figure 2A). Furthermore, the ADF intake during the pre-weaning period was respectively 1.6 and 2.9 times higher in groups G and A than C (Figure 2B). Obviously, the intake of the other fiber components changed in the three groups, considering that the fiber components (NDF, ADL, cellulose, and hemicellulose) were different in grass, dehydrated alfalfa, and control diets.

The feed and fiber intakes of the rabbits after weaning (30–45 days) were more similar than during milking; however, the A and G groups generally showed significantly higher values (Table 2).

### 3.3. Caecal Microbiota Analysis

**Figure A1:**
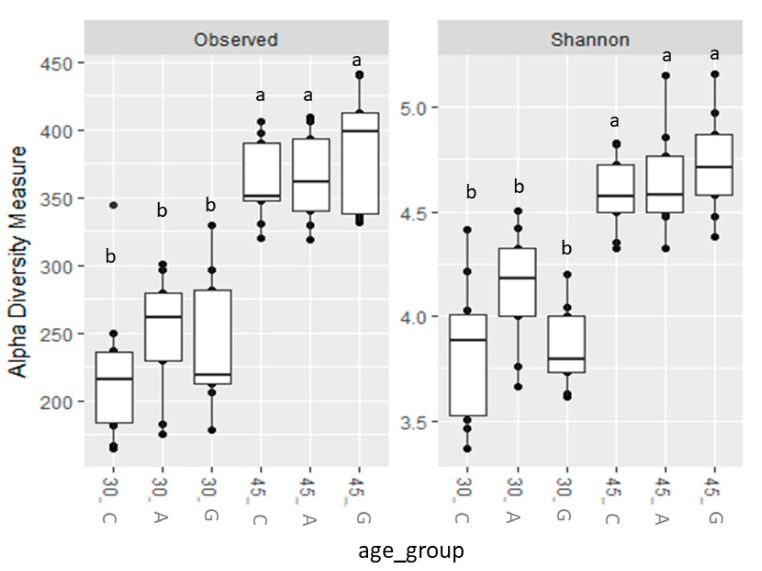
Effect of dietary supplementation with dehydrated alfalfa or fresh grass in peri-weaning period on alpha-diversity rabbit caecal microbiota: (**left**) observed means species richness; (**right**) Shannon diversity. C = rabbit fed pelleted feed only; A = rabbit fed pelleted feed + dehydrated alfalfa; G = rabbit fed pelleted feed + fresh grass. ^a,b^ Values within a row with different superscript letters differ significantly at *p* < 0.05.

**Figure A2:**
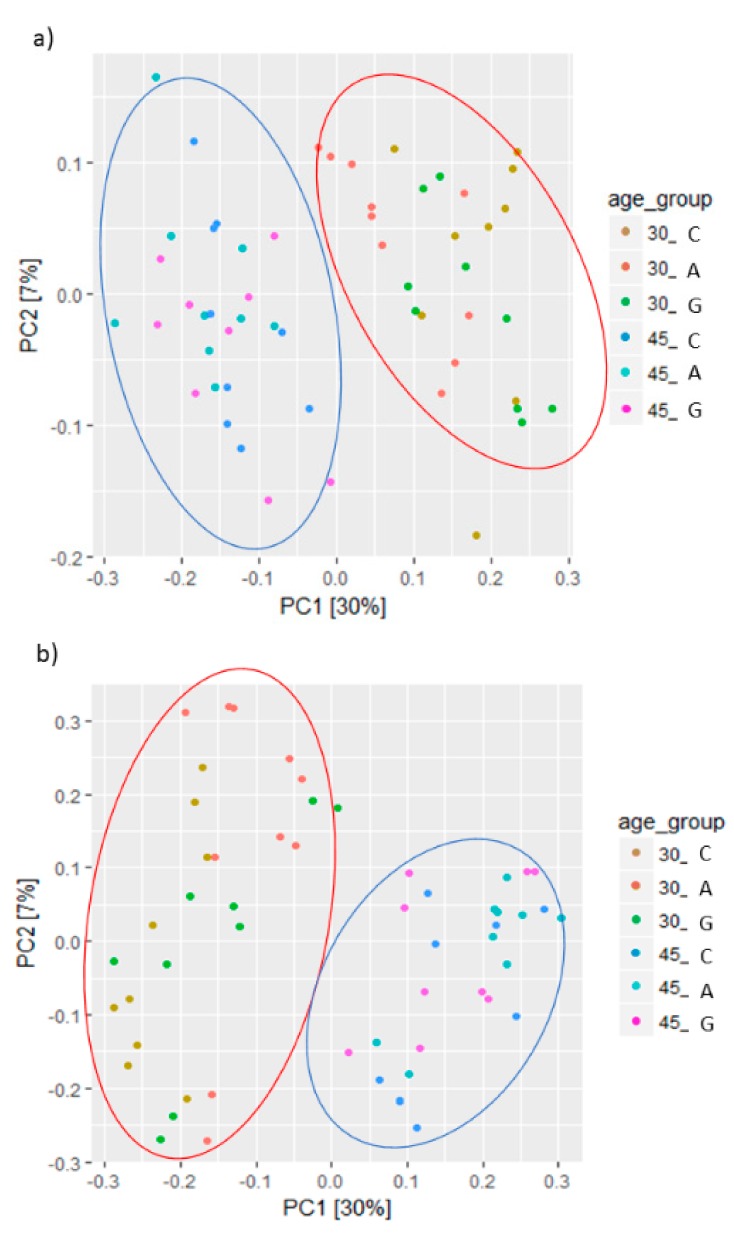
MDS plots of the rarefy weighted UniFrac distances (**a**) and Bray–Curtis distances (**b**) of caecal microbiota of rabbit fed a dietary supplementation of dehydrated alfalfa or fresh grass in peri-weaning period (those at 30 and 45 days of age were analyzed). Principal component (PC)1 and PC2 values for each sample are plotted; the percentage variation explained by each PC is shown in brackets. C = rabbit fed pelleted feed only; A = rabbit fed pelleted feed + dehydrated alfalfa; G = rabbit fed pelleted feed + fresh grass.

The alpha diversity (Figure A1 in Appendix A), indicated by the Shannon index, showed a significant difference between the two ages; at 30 days of age, the observed OTUs were lower than at 45 days, but there was no significant change (*p* > 0.05) due to the feeding strategyFigure 3 and Figure A2 (Appendix A) show the plots representing the caecal microbiota diversity in relation to the feeding strategy and age. At 30 and 45 days, the groups were separate in both multidimensional scaling (MDS; principal coordinate analysis) plots generated based on the UniFrac (Figure A2a) and Bray–Curtis distance (Figure A2b).

Analysis of the taxonomic composition of the bacterial community revealed that, at 45 days, the phyla profile was very different to that at 30 days. At both 30 and 45 days of age, Firmicutes was the predominant phylum; however, at 30 days, Bacteroidetes had a higher percentage than at 45 days.

**Figure 4:**
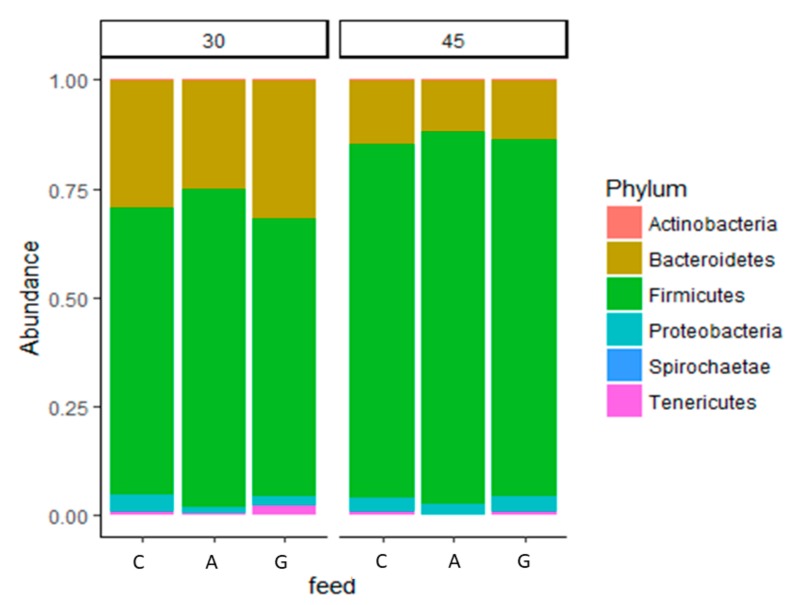
Effect of dietary supplementation with dehydrated alfalfa or fresh grass on peri-weaning period on relative abundances (%) of the different phyla of rabbit caecal microbiota at 30 and 45 days of age. C = rabbit fed pelleted feed only; A = rabbit fed pelleted feed + dehydrated alfalfa; G = rabbit fed pelleted feed + fresh grass.

**Figure 5:**
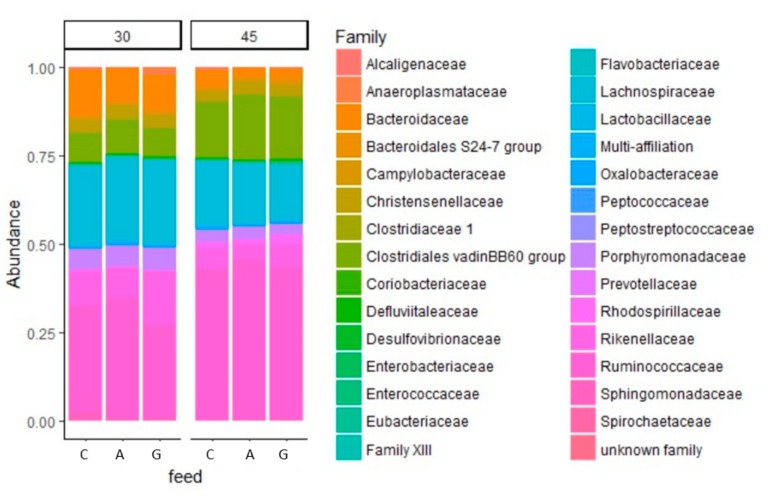
Effect of dietary supplementation with dehydrated alfalfa or fresh grass on peri-weaning period on relative abundances (%) of the different families of rabbit caecal microbiota at 30 and 45 days of age. C = rabbit fed pelleted feed only; A = rabbit fed pelleted feed + dehydrated alfalfa; G = rabbit fed pelleted feed + fresh grass.

Analysis of the abundance of the phyla and families of the bacterial community (Figure 4 and Figure 5) revealed many differences between the diets (Table 3). The A groups at 30 days of age showed a higher proportion of Firmicutes than the C and G groups (72.07 vs. 65.78 and 62.38%, respectively), which was also shown by the Firmicutes/Bacteroidetes ratio (2.90 vs. 2.34 and 2.05 in groups A, C, and G, respectively). Both dietary supplemented groups showed a lower percentage of Protobacteria at 30 days than the control.

The Ruminococcaceae/Lachinospiraceae ratio was the highest in the A group at both ages, followed by the G group then the C one (8.67 vs. 0.13 and 0.08% at 30 days and 1.99 vs. 0.19 and 0.07% at 45 days, respectively, for the G and C groups). In particular, the most abundant families in the samples were Lachnospiraceae, Ruminococcaceae, and Clostridiales with more than 5%, followed by Lactobacillaceae, Coriobacteriacee, and Bacteroidaceae.

## 4. Discussion

Rabbits, being cecotrophic mammals, have a microbiota which differs from other herbivorous species and provides approximately 30–50% of the maintenance energy requirements of an adult [26]. However, the transition from milk to solid feed, like in other mammals, is a very critical period for the establishment of a proper and durable microbiota [13,15]. The microbiota depends on the feed provided, since it acts as the substrate for microorganisms, modulating the physico-chemical conditions of the microbial community, intestinal motility, and digestive transit [15]. Apart from feed composition, the initial age of solid feed intake also has a significant impact on the development and maturity of the digestive tract of young rabbits [15].

In the present study, we demonstrated that fiber-rich dietary supplies (dehydrated alfalfa and, to a lesser degree, fresh grass) furnished in the early-life period orients the digestive ecosystem of young rabbits, without any negative effect on productive performance. Our results showed that groups G and A increased feed intake during the pre-weaning period (15–21 days: 3.6 vs. 8.6 and 14.2 g/day/rabbit, respectively, in groups C, A, and G) with respect to the control. Fresh grass and alfalfa also increased fiber intake (e.g., ADF 1.2- and 2.5-fold; cellulose 1.6- and 3.0-fold, respectively).

Our previous study, which used fresh chicory as supplemental feed, showed a similar trend [27]; however, the caecal content of rabbit fed chicory had a lower dry matter, because of the higher moisture of fresh vegetable.

Mousa et al. [28] administered different tropical green forage to rabbits and observed an increase in feed intake, even if this was not followed by any improvement in body weight. Other researchers showed that feed intake by litters generally starts at about 17 days of age and is very low in the first days (2.5 g/day between 18 and 21 days [29]) with a substantial increase one week later [30]. Accordingly, cecotrophia starts some days later, and its activity is associated with a stable and higher feed intake [9].

The role of dietary fiber in rabbit digestive physiology was deeply studied [31,32,33], and the role of different fiber classes (i.e., NDF, hemicelluloses, pectins, ADL, ADF, and cellulose) was assessed [34,35,36]. Maitre et al. [36] stated that the supply of lignocellulose (ADF) favors digestive disorders and mortality in fattening rabbits, and a high value of ADF (>25%) is also associated with a slow growth rate [35]. Furthermore, a linear relationship between ADL value and mortality (by diarrhea) in young rabbits was reported, since a higher lignin content is associated with a lower retention time of *digesta* in the intestinal tract (−20%) [37]. Accordingly, Gidenne [10] suggested the use of a minimum quantity of lignocellulose (ADF, 190 g/kg of raw feed) and lignin (ADL, 55 g/kg of raw feed) in the diet of young rabbits (until 45 days of age).

Many differences were found in the microbiota structure of different aged rabbits. The caecal microbiota of young rabbits during the lactation period (15–30 days) strongly differed from that of post-weaning rabbits (45 days). Firmicutes was the most abundant phylum (~74% of the total phyla), which reached higher values at 45 days, whereas Bacteriodetes, which was the second most abundant phylum (~13%), showed the opposite trend in all the experimental groups. Other phyla constituted the less abundant portion of the bacterial community (<5%) and showed a higher within-group variability. In addition, we found a predominance of Lachnospiraceae.

It should be noted that the higher variability observed in the microbiota within dietary groups could be related to the relative gut immaturity of young rabbits. Similarly, Combes et al. [13] found a progressive reduction in the distances between the bacterial communities of two consecutive ages from the neonatal period to the sub-adult period, indicating a progressive stability up to 70 days of age (the period in which there is homogenization). The authors reported that the total bacterial copy number reached a maximum at 21 days; however, the changes were widely dependent on phyla; the Bacteroides/Prevotella increased from 14 to 21 days, remained stable until 35 days, and decreased at 70 days to a level similar to that at 14 days, whereas the Firmicutes remained stable between 14 and 70 days.

In agreement, Padilha et al. [38] reported that the microbiota of young rabbits changed after feed transition (from milk to dry feed) at around three weeks of age.

At weaning, the caecal ecosystem of litters is not yet fully developed and stabilized [37]; therefore, the microbiota of the mother also plays a key role in establishing that of the litters. Coprophagia by young rabbits was described by Combes and co-workers [9], and it is an important behavior for the establishment of a proper intestinal microbiota. Zhang et al. [39] reported that the doe–litter separation during suckling (probably associated with no or lower coprophagia) reduced the intestinal bacteria richness and negatively affected the development of the intestinal digestive and immune systems and the growth performance of litters.

The observed proportion of phyla agreed with that reported by Bäuerl et al. [40], whereas Combes et al. [9] found a higher proportion of Firmicutes (94%) and lower proportion of Bacteriodetes (12%) in adult rabbits. It is likely, as already stated, that some discrepancies are related to the different ages of the rabbits; indeed, in our experiment, the bacteria proportions changed with age.

The main phyla which constituted the rabbit microbiota are described as capable of degrading cellulose, in the order of efficiency of Firmicutes > Actinobacteria > Proteobacteria > Bacteroidetes [41]. In agreement, a possible shift in the microbiota profile found on A group with respect to G and C (Firmicutes > Bacteroidetes) was likely due to the higher amount of cellulose or lignocellulose ingested, as demonstrated by present results. Such changes were more effective at 45 days of age, when the cellulose intake was higher than that at 30 days (17.53 vs. 15.75 and 15.05 at 45 days and 3.49 vs. 1.83 and 1.15 at 30 days, respectively, in A, G, and C).

Bacteroidetes are believed to be one of the major commensal phyla in the gut of rabbits and they were proven to stimulate the development of gut-associated immune tissue [42,43]. Accordingly, the Firmicutes/Bacteroidetes ratio showed a significant difference due to the age and feed. Mariat et al. [44], reported that the amount of Bacteroidetes is generally higher in the intestine of young healthy rabbits than in adults, and, after 56 days, the shift is almost definitive.

In our study, the proportion of Firmicutes at 30 days of age was highest in the A group and had a value similar to that at 45 days, whereas group G had the lowest percentage of this phylum and a higher proportion of Bacteroidetes than the control. Such results suggested a better fiber digestion by the A group; in agreement, Fortun-Lamothe et al. [45] reported that the bacteria involved in fibrolysis (hydrolysis of cellulose, xylanes, pectins, etc.) become established only when intake of solid feed begins and when a fibrous substrate enters the caecum. Probably, the early administration of a two-fold higher quantity of lignocellulose (ADF values until 30 days) in the A group with respect to the G group better arranged the intestine bacteria community to its use. Furthermore, alfalfa administration also changed the microbiota in terms of family composition: increasing the Ruminococcaceae/Lachnospiraceae ratio compared to the control group. In agreement, Combes and co-workers [9] described the Ruminococcaceae/Lachnospiraceae ratio as an important marker for intestinal health; higher values of Lachnospiraceae were found in healthy young rabbits due to the stimulation of cecotrophic behavior, which was also associated with a reduction in mortality. *Ruminococcus* is the most relevant genus of the Firmicutes phylum that is dominant in healthy rabbits and notably decreases in the presence of disease [46,47].

## 5. Conclusions

Although the main effects on the rabbit microbiota were related to age, taken together, these results suggest that different dietary supplies can strongly modify the microbiota and could be a helpful strategy to promote the health of young rabbits during the peri-weaning period. The use of dehydrated alfalfa seems to be more effective than fresh grass, mainly because of the higher fiber intake. However, further studies are needed to better understand the changes, in terms of species and/or functional microorganisms, in the composition of the targeted microbiota, as well as the role of these changes in the immune system and, consequently, the health of young rabbits.

## Figures and Tables

**Figure 3:**
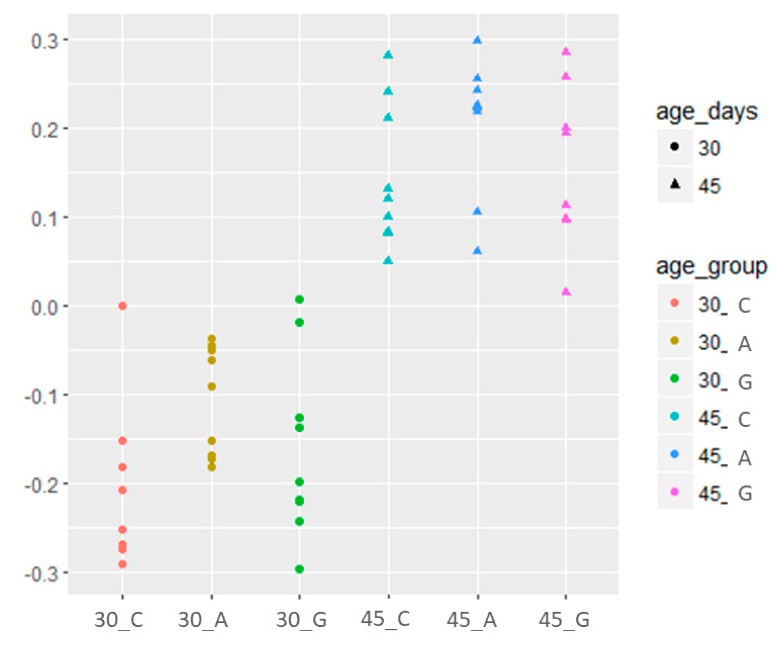
Effect of dietary supplementation with dehydrated alfalfa or fresh grass on peri-weaning period of rabbit caecal microbiota beta diversity. First axis of multidimensional scaling (MDS) ordination based on the Bray–Curtis distance matrix plotted against days of treatment. C = rabbit fed pelleted feed only; A = rabbit fed pelleted feed + dehydrated alfalfa; G = rabbit fed pelleted feed + fresh grass.

**Table 1:** Proximate composition of rabbit feed, dehydrated alfalfa, and fresh grass (g/100 g of dry matter).

Proximate Composition	Pelleted Feed	Dehydrated Alfalfa	Fresh Grass
Dry Matter	89.00	88.00	35.61
Crude protein	20.45	13.01	11.57
Ether extract	4.63	2.09	2.50
Ash	11.57	8.06	25.02
Crude fiber	26.99	38.01	30.61
Neutral detergent fiber (NDF)	24.02	51.75	25.46
Acid detergent fiber (ADF)	14.12	38.72	15.77
Acid digestible lignin (ADL)	2.91	7.10	3.22
Hemicellulose	9.91	13.03	9.70
Cellulose	11.21	31.72	12.55

**Table 2:** Effect of dehydrated alfalfa or fresh grass supplementation on productive performance of rabbit does and young rabbits.

Productive Performances	Unit	Experimental Groups ^1^			Sig. ^2^
		C	A	G	
Rabbit born per doe	*n* litters	9	9	9	-
Weaned rabbits (30 days)	*n*	8	8	8	ns
Rabbits at 45 days	“	8	8	8	ns
Individual weight at birth	g	53.10 ± 3.10	55.12 ± 2.34	51.34 ± 2.50	ns
Individual weight at 15 days	“	240.0 ± 8.2	243.00 ± 5.78	239.30 ± 7.12	ns
Individual weight at 30 days	“	489.0 ± 121.5	500.50 ± 136.59	485.50 ± 74.23	ns
Individual weight at 45 days	“	1058.00 ± 83.104	1050.44 ± 138.17	1019.1 ± 145.71	ns
Daily weight gain 0–15 days	g/day/rabbit	12.46 ± 3.12	12.53 ± 2.87	12.53 ± 1.16	ns
Daily weight gain 15–30 days	“	16.6 ± 5.32	17.13 ± 6.87	16.4 ± 8.81	ns
Daily weight gain 30–45 days	“	37.93 ± 10.22	36.66 ± 6.66	35.60 ± 4.65	ns
Pre-weaning mortality (0–30 days)	%	12.5	12.5	12.5	ns
Milk production 0–15 days	g/day/doe	148.02 ± 11.83	143.34 ± 10.54	145.35 ± 11.86	ns
Milk intake 0–15 days	g/day/rabbit	18.50 ± 2.16	17.91 ± 3.54	17.79 ± 1.86	ns
Pelleted feed intake 15–30 days	“	11.55 ± 1.02 ^a^	6.56 ± 0.53 ^b^	7.43 ± 0.65 ^b^	*
Supplemental intake 15–30 days	“	-	10.18 ± 4.25 ^b^	16.72 ± 3.99 ^a^	*
Total feed intake (15–30 days)	“	11.55 ± 1.02 ^c^	16.74 ± 4.18 ^b^	24.15 ± 3.87 ^a^	*
NDF intake (15–30 days)	“	2.47 ± 0.33 ^c^	6.04 ± 0.32 ^a^	3.09 ± 0.14 ^b^	*
ADF intake (15–30 days)	“	1.45 ± 0.21 ^c^	4.29 ± 0.26 ^a^	2.38 ± 0.13 ^b^	*
ADL intake (15–30 days)	“	0.29 ± 0.04 ^c^	1.05 ± 0.04 ^a^	0.38 ± 0.01 ^b^	*
Cellulose intake (15–30 days)	“	1.15 ± 0.12 ^c^	3.49 ± 0.19 ^a^	1.83 ± 0.06 ^b^	*
Pelleted feed intake 30–45 days	“	150.81 ± 56.28 ^a^	143.26 ± 57.60 ^b^	146.45 ± 32.62 ^ab^	*
Supplemental intake 30–45 days	“	-	11.56 ± 3.68 ^b^	25.12 ± 2.55 ^a^	*
Feed efficiency		3.98	3.90	4.10	ns
NDF intake (30–45 days)	g/day/rabbit	32.24 ± 2.15 ^b^	35.92 ± 2.89 ^a^	33.61 ± 2.84 ^b^	*
ADF intake (30–45 days)	“	18.95 ± 1.08 ^c^	21.96 ± 1.31 ^a^	19.83 ± 1.07 ^b^	*
ADL intake (30–45 days)	“	3.90 ± 0.34 ^b^	4.43 ± 0.40 ^a^	4.08 ± 0.01 ^b^	*
Cellulose intake (30–45 days)	“	15.04 ± 1.00 ^b^	17.53 ± 0.97 ^a^	15.75 ± 0.98 ^b^	*

^1^ C = rabbit fed pelleted feed only; A = rabbit fed pelleted feed and dehydrated alfalfa; G = rabbit fed pelleted feed and fresh grass. ^2^ Sig. = significance; ns = not significant. ^a,b,c^ Values within a row with different superscript letters differ significantly at *p* < 0.05.

**Table 3:** Effect of dietary supplementation with dehydrated alfalfa or fresh grass on peri-weaning period on relative abundances (%) of the different phyla and on Ruminococcaceae/Lachnospiraceae family ratio and Firmicutes/Bacteroidetes ratio of rabbit caecal microbiota at 30 and 45 days.

Phyla	Experimental Groups ^1^						CV ^2^	*p* ^3^	
	30 Days			45 Days					
	C	A	G	C	A	G		Age	Feed
Firmicutes	65.78 ^c^	72.07 ^ab^	62.38 ^c^	78.87 ^b^	83.64 ^a^	79.98 ^b^	6.65	***	*
Bacteroidetes	28.15 ^a^	24.83 ^a^	30.41 ^a^	14.15 ^b^	12.75 ^b^	13.26 ^b^	4.32	***	ns
Proteobacteria	4.50 ^a^	1.59 ^b^	1.92 ^b^	3.02 ^ab^	2.30 ^ab^	3.48 ^ab^	1.04	*	*
Tenericutes	0.76 ^b^	1.19 ^b^	4.22 ^a^	3.02 ^a^	1.10 ^b^	2.28 ^ab^	0.82	*	ns
Actinobacteria	0.78 ^ab^	0.29 ^b^	1.05 ^a^	0.85 ^ab^	0.17 ^b^	0.96 ^ab^	0.63	*	ns
Spirochaetae	0.03	0.03	0.02	0.09	0.04	0.04	0.05	ns	ns
Firmicutes/Bacteroidetes	2.34 ^d^	2.90 ^c^	2.05 ^d^	5.57 ^b^	6.56 ^a^	6.03 ^ab^	1.02	**	*
Ruminococcaceae/Lachnospiraceae	<0.001 ^d^	1.58 ^b^	0.03 ^d^	0.77 ^c^	24.32 ^a^	0.07 ^d^	3.12	**	*

^1^ C = rabbit fed pelleted feed only; A = rabbit fed pelleted feed + dehydrated alfalfa; G = rabbit fed pelleted feed + fresh grass. ^2^ CV = coefficient of variation. ^3^ ns = not significant. ^a–d^ Values within a row with different superscript letters differ significantly as followed reported: *** *p* < 0.0001, ** *p* < 0.001, * *p* < 0.01.

## References

[1] Sekirov I., Russell S.L., Antunes L.C.M., Finlay B.B. (2010). Gut microbiota in health and disease. Physiol. Rev..

[2] Maertens L., Falcao-e-Cunha L., Marounek M. (2006). Feed additives to reduce the use of antibiotics. Recent Advances in Rabbit Sciences.

[3] Nogacka A.M., Salazar N., Arboleya S., Suárez M., Fernández N., Solís G., de Los Reyes-Gavilán C.G., Gueimonde M. (2018). Early microbiota, antibiotics and health. Cell. Mol. Life Sci..

[4] Berendonk T.U., Manaia C.M., Merlin C., Fatta-Kassinos D., Cytryn E., Walsh F., Bürgmann H., Sørum H., Norström M., Pons M.N. (2015). Tackling antibiotic resistance: The environmental framework. Nat. Rev. Microbiol..

[5] Argañaraz-Martínez E., Babot J.D., Apella M.C., Chaia A.P. (2013). Physiological and functional characteristics of Propionibacterium strains of the poultry microbiota and relevance for the development of probiotic products. Anaerobe.

[6] Lan Y., Verstegen M.W.A., Tamminga S., Williams B.A. (2005). The role of the commensal gut microbial community in broiler chickens. World’s Poultry Sci. J..

[7] Leser T.D., Amenuvor J.Z., Jensen T.K., Lindecrona R.H., Boye M., Møller K. (2002). Culture-independent analysis of gut bacteria: The pig gastrointestinal tract microbiota revisited. Appl. Environ. Microbiol..

[8] Combes S., Fortun-Lamothe L., Cauquil L., Gidenne T. (2013). Engineering the rabbit digestive ecosystem to improve digestive health and efficacy. Animal.

[9] Combes S., Gidenne T., Cauquil L., Bouchez O., Fortun-Lamothe L. (2014). Coprophagous behavior of rabbit pups affects implantation of cecal microbiota and health status. J. Anim. Sci..

[10] Gidenne T. (2010). Recent advances in rabbit nutrition: Emphasis on fibre requirements. A review. World Rabbit Sci..

[11] Carabaño R., Navarro I.B., Chamorro S., García J., Ruiz A.G. (2008). New trends in rabbit feeding: Influence of nutrition on intestinal health. Span. J. Agric. Res..

[12] Michelland R.J., Combes S., Monteils V., Cauquil L., Gidenne T., Fortun-Lamothe L. (2010). Molecular analysis of the bacterial community in digestive tract of rabbit. Anaerobe.

[13] Combes S., Michelland R.J., Monteils V., Cauquil L., Soulié V., Tran N.U., Gidenne T., Fortun-Lamothe L. (2011). Postnatal development of the rabbit caecal microbiota composition and activity. FEMS Microbiol. Ecol..

[14] Abecia J.A., Sosa C., Forcada F., Meikle A. (2006). The effect of undernutrition on the establishment of pregnancy in the ewe. Reprod. Nutr. Dev..

[15] Gidenne T., Combes S., Fortun-Lamothe L. (2012). Feed intake limitation strategies for the growing rabbit: Effect on feeding behaviour, welfare, performance, digestive physiology and health: A review. Animal.

[16] Gallois M., Gidenne T., Fortun-Lamothe L., Le Huerou-Luron I. (2005). An early stimulation of solid feed intake slightly influences the morphological gut maturation in the rabbit. Reprod. Nutr. Dev..

[17] Tumova E., Zita L., Skrivanova V., Fucikova A., Skrivan M., Buresova M. (2007). Digestibility of nutrients, organ development and blood picture in restricted and ad libitum fed broiler rabbits. Archiv. Fur Geflugelkunde.

[18] Association of Official Analytical Chemists (AOAC) (1995). Official Methods of Analysis.

[19] Van Soest P.V., Robertson J.B., Lewis B.A. (1991). Methods for dietary fiber, neutral detergent fiber, and nonstarch polysaccharides in relation to animal nutrition. J. Dairy Sci..

[20] Escudié F., Auer L., Bernard M., Mariadassou M., Cauquil L., Vidal K., Maman S., Hernandez-Raquet G., Combes S., Pascal G. (2018). FROGS: Find, rapidly, OTUs with galaxy solution. Bioinformatics.

[21] Mahé F., Rognes T., Quince C., de Vargas C., Dunthorn M. (2014). Swarm: Robust and fast clustering method for amplicon-based studies. Peer J..

[22] Quast C., Pruesse E., Yilmaz P., Gerken J., Schweer T., Yarza P., Peplies J., Glöckner F.O. (2013). The silva ribosomal RNA gene database project: Improved data processing and web-based tools. Nucleic Acids Res..

[23] StataCorp (2015). Stata Statistical Software: Release 14.0.

[24] R Core Team (2017). R: A Language and Environment for Statistical Computing.

[25] RStudio Team (2016). Rstudio: Integrated Development for R.

[26] Bagóné Vántus V., Kovács M., Zsolnai A. (2014). The rabbit caecal microbiota: Development, composition and its role in the prevention of digestive diseases—A review on recent literature in the light of molecular genetic methods. Acta Agraria Kaposvan Riensis.

[27] Castellini C., Cardinali R., Rebollar P.G., Dal Bosco A., Jimeno V., Cossu M.E. (2007). Feeding fresh chicory (Chicoria intybus) to young rabbits: Performance, development of gastro-intestinal tract and immune functions of appendix and Peyer’s patch. Anim. Feed Sci. Technol..

[28] Mousa M.R.M. (2011). Effect of feeding acacia as supplements on the nutrient digestion, growth performance, carcass traits and some blood constituents of Awassi lambs under the conditions of North Sinai. Asian J. Anim. Sci..

[29] Read T., Combes S., Gidenne T., Destombes N., Grenet L., Fortun-Lamothe L. (2015). Stimulate feed intake before weaning and control intake after weaning to optimise health and growth performance. World Rabbit Sci..

[30] Orengo J., Gidenne T. (2007). Feeding behaviour and caecotrophy in the young rabbit before weaning: An approach by analysing the digestive contents. Appl. Anim. Behav. Sci..

[31] Gidenne T., Garcia J. (2006). Nutritional strategies improving the digestive health of the weaned rabbit. Recent Advances in Rabbit Sciences.

[32] Gidenne T., Debray L., Fortun-Lamothe L., Le Huërou-Luron I. (2007). Maturation of the intestinal digestion and of microbial activity in the young rabbit: Impact of the dietary fibre: Starch ratio. Comp. Biochem. Physiol. Part A Mol. Integr. Physiol..

[33] Xiccato G., Trocino A., Majolini D., Fragkiadakis M., Tazzoli M. (2011). Effect of decreasing dietary protein level and replacing starch with soluble fibre on digestive physiology and performance of growing rabbits. Animal.

[34] Perez J.M., Gidenne T., Bouvarel I., Arveux P., Bourdillon A., Briens C., Mirabito L. (2000). Replacement of digestible fibre by starch in the diet of the growing rabbit. II. Effects on performances and mortality by diarrhoea. Annales de Zootech.

[35] Gidenne T., Arveux P., Madec O. (2001). The effect of the quality of dietary lignocellulose on digestion, zootechnical performance and health of the growing rabbit. Anim. Sci..

[36] Maitre I., Lebas F., Arveux P., Bourdillon Supperay J., Saint Cast Y. Taux de lignocellulose (ADF de Van Soest) et performances de croissance du lapin de chair. Proceedings of the 5Èmes Journées de la Recherche Cunicoles.

[37] Gidenne T. (1997). Caeco-colic digestion in the growing rabbit: Impact of nutritional factors and related disturbances. Livest. Prod. Sci..

[38] Padilha M.T.S., Licois D., Gidenne T., Carrè B., Fonty G. (1995). Relationships between microflora and caecal fermentation in rabbits before and after weaning. Reprod. Nutr. Dev..

[39] Zhang Y., Cui H., Sun D., Liu L., Xu X. (2018). Effects of doe-litter separation on intestinal bacteria, immune response and morphology of suckling rabbits. World Rabbit Sci..

[40] Bäuerl C., Collado M.C., Zúñiga M., Blas E., Martínez G.P. (2014). Changes in cecal microbiota and mucosal gene expression revealed new aspects of epizootic rabbit enteropathy. PLoS ONE.

[41] Rhee K.J., Sethupathi P., Driks A., Lanning D.K., Knight K.L. (2004). Role of commensal bacteria in development of gut-associated lymphoid tissues and preimmune antibody repertoire. J. Immunol..

[42] Hanson N.B., Lanning D.K. (2008). Microbial induction of B and T cell areas in rabbit appendix. Dev. Comp. Immunol..

[43] Mariat D., Firmesse O., Levenez F., Guimarăes V.D., Sokol H., Doré J., Corthier G., Furet J.P. (2009). The Firmicutes/Bacteroidetes ratio of the human microbiota changes with age. BMC Microbiol..

[44] Koeck D.E., Pechtl A., Zverlov V.V., Schwarz W.H. (2014). Genomics of cellulolytic bacteria. Curr. Opin. Biotechnol..

[45] Fortun-Lamothe L., Boullier S. (2007). A review on the interactions between gut microflora and digestive mucosal immunity. Possible ways to improve the health of rabbits. Livest. Sci..

[46] Morrow A., Lagomarcino A., Schibler K., Taft D., Yu Z., Wang B., Altaye M., Wagner M., Gevers D., Ward D.V. (2013). Early microbial and metabolomic signatures predict later onset of necrotizing enterocolitis in preterm infants. Microbiome.

[47] Comito D., Romano C. (2012). Dysbiosis in the pathogenesis of pediatric inflammatory bowel diseases. Int. J. Inflamm..

